# Comparative *in-silico* analysis of microbial dysbiosis discern potential metabolic link in neurodegenerative diseases

**DOI:** 10.3389/fnins.2023.1153422

**Published:** 2023-04-11

**Authors:** Vipin Chauhan, Nitin K. Chauhan, Somit Dutta, Dhruv Pathak, Upendra Nongthomba

**Affiliations:** ^1^Developmental and Biomedical Genetics Laboratory, Department of Molecular Reproduction, Development and Genetics, Indian Institute of Science, Bengaluru, India; ^2^School of Computational and Integrative Science, Jawaharlal Nehru University, New Delhi, India

**Keywords:** Alzheimer's disease, Parkinson's disease, Multiple sclerosis, Amyotrophic lateral sclerosis, microbial dysbiosis, neuro-immunomodulatory compound, neurotransmitter, neuroprotective compound

## Abstract

A healthy gut flora contains a diverse and stable commensal group of microorganisms, whereas, in disease conditions, there is a shift toward pathogenic microbes, termed microbial dysbiosis. Many studies associate microbial dysbiosis with neurodegenerative diseases, including Alzheimer's disease (AD), Parkinson's disease (PD), Multiple sclerosis (MS), and Amyotrophic lateral sclerosis (ALS). Although, an overall comparative analysis of microbes and their metabolic involvement in these diseases is still lacking. In this study, we have performed a comparative analysis of microbial composition changes occurring in these four diseases. Our research showed a high resemblance of microbial dysbiosis signatures between AD, PD, and MS. However, ALS appeared dissimilar. The most common population of microbes to show an increase belonged to the phyla, *Bacteroidetes, Actinobacteria, Proteobacteria*, and *Firmicutes*. Although, *Bacteroidetes* and *Firmicutes* were the only phyla that showed a decrease in their population. The functional analysis of these dysbiotic microbes showed several potential metabolic links which can be involved in the altered microbiome-gut-brain axis in neurodegenerative diseases. For instance, the microbes with elevated populations lack pathways for synthesizing SCFA acetate and butyrate. Also, these microbes have a high capacity for producing L-glutamate, an excitatory neurotransmitter and precursor of GABA. Contrastingly, Tryptophan and histamine have a lower representation in the annotated genome of elevated microbes. Finally, the neuroprotective compound spermidine was less represented in elevated microbes' genomes. Our study provides a comprehensive catalog of potential dysbiotic microbes and their metabolic involvement in neurodegenerative disorders, including AD, PD, MS, and ALS.

## 1. Introduction

**Figure 1:**
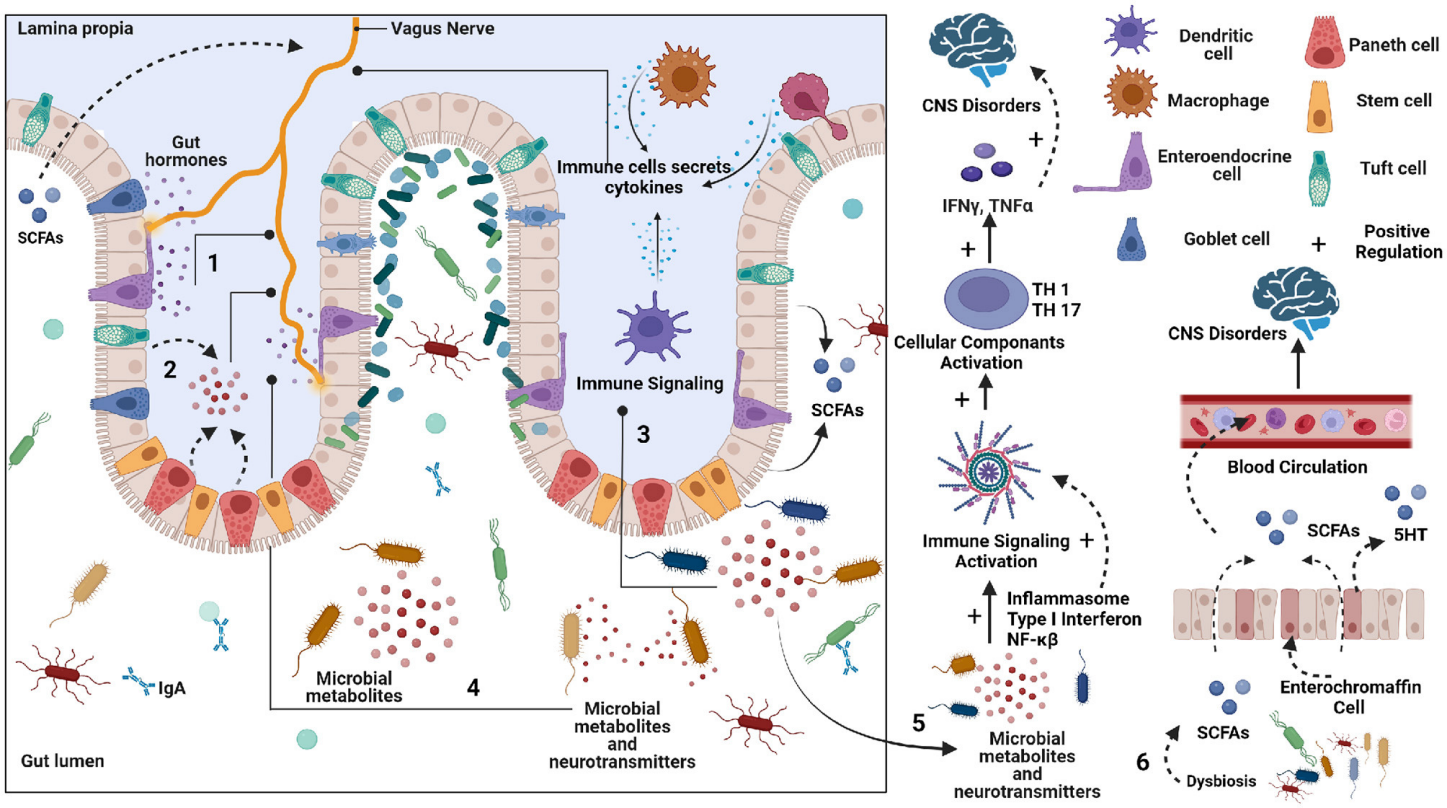
Schematic representation of potential pathways by which gut can affect the Central nervous system (CNS). 1. Enteroendocrine cells (EECs) secreted various peptides/amines can affect vagus nerve signaling to the brain. 2. Neurotransmitter precursors and metabolites are formed by gut microbes that circulate to the brain. Some bypass the blood-brain barrier. 3. The pathogenic microbes can elicit an immune response, which can produce cytokines that circulate from the blood to the brain. 4. Microbial metabolites and neurotransmitters can stimulate an Enteric nervous system (ENS). 5. The Neurotransmitter precursors and metabolites produced by microbes can alter brain physiology. 6. The short chain fatty acid (SCFA) produced by gut microbes can affect brain function.

We have seen the expanding numbers of neurodegenerative diseases in the last 25 years globally (Feigin et al., 2017). Among all the neurological disorders, neurodegenerative diseases like Alzheimer's disease (AD), Parkinson's disease (PD), and Multiple sclerosis (MS) were responsible for 20.3%, 2%, and 0.2% of deaths, respectively (Feigin et al., 2017). According to a prediction by WHO, cases of neurodegenerative diseases will surpass the number of cancer cases worldwide by 2040 (Feigin et al., 2017). In the past decade, extensive work on the microbiome-gut-brain axis has put forward the gut microbes as potential candidates to look for in neurodegenerative diseases. Studies show a decrease in microbial diversity and microbial dysbiosis in diseases including AD, PD, and MS and ALS (Sarkar and Banerjee, 2019). Gut microbes and the brain communicate bidirectionally through various neuroendocrine, neuroimmune and neural connections like the vagus nerve (Peterson, 2020). Gut microbes can affect brain functions by producing neurotransmitters and precursors such as GABA (γ-aminobutyric acid), dopamine, serotonin, and noradrenaline. Certain microorganisms, including *Bifidobacterium, Escherichia* spp., *Bacteroides*, and *Parabacteroides*, produce GABA, which is an inhibitory neurotransmitter (Strandwitz et al., 2019). Gut microbes also play a critical role in serotonin production. Interestingly, microbial metabolites such as α-tocopherol, tyramine, p-aminobenzoate, and indole impact serotonin 5-HT secretion by enteroendocrine cells in the intestine (Figure 1) (Yano et al., 2015; Morris et al., 2017). Studies show that gut microbiota can affect brain function through direct and indirect signaling. Short-chain fatty acids (SCFA) are suggested as key molecules in microbiota-gut-brain interaction (Cummings et al., 1987). Gut microbes produce SCFA by anaerobic fermentation of indigestible polysaccharides, including dietary fiber and resistant starch. The acetate (C2), propionate (C3), and butyrate (C4) are the main SCFA produced in the intestine. After the SCFA are utilized as an energy source by colonocytes in the intestine (**Figure 5A**). They are also shown to regulate the intestinal barrier and influence the inflammatory response (Ardawi and Newsholme, 1985; Cummings et al., 1987; Inan et al., 2000; Kelly et al., 2015; Kaiko et al., 2016). In rat and primary microglial cell cultures, the addition of acetate decreases pro-inflammatory cytokines and microglia activation (Soliman et al., 2012; Smith et al., 2014). Also, It is known that butyrate induces the breakdown of α-synuclein and enhances remyelination in mice cerebellar slice cultures (Chen et al., 2019; Qiao et al., 2020). In a mouse model of Alzheimer's disease, increased gut microbiota-produced propionate correlates with neuroinflammation and cognitive decline (Cuervo-Zanatta et al., 2020). Additionally, dysbiotic gram-negative bacteria in AD and PD may induce the generation of pro-inflammatory cytokines and inflammatory response through Lipopolysaccharides (LPSs) (Itzhaki et al., 2016; Ransohoff, 2016; Sarkar and Banerjee, 2019). The production of these molecules disrupts the intestinal barrier, which causes enhanced systemic circulation of microbial products and inflammatory factors. Consequently, the inflammatory factors that disrupt the blood-brain barrier may result in neuroinflammation (Gammon, 2014; Heneka et al., 2015; Sarkar and Banerjee, 2019). Recent studies have indicated that gut microbial communities play a vital role in regulating innate and adaptive immune homeostasis in the host. Immune dysregulation brought on by changes to these gut microbial communities can result in autoimmune diseases such as MS (Wu and Wu, 2012; Ochoa-Repáraz et al., 2018; Jiao et al., 2020). The pathological hallmark of MS is defined by focal demyelinated plaques within the central nervous system (CNS), with gliosis, inflammation and neurodegeneration (Popescu et al., 2013). The gut microbial dysbiosis in MS leads to the reduction of microbes which can elicit an anti-inflammatory response through immune cells like T regulatory cells, regulatory B cells and Interleukin 10 (IL-10) in host (Shahi et al., 2017). On the other hand, microbes which are found in abundance can induce a pro-inflammatory response in the host through dendritic and B cells (Chung and Kasper, 2010; Shahi et al., 2017). The ALS mouse model G93A SOD1 (superoxide dismutase) is shown to have increased intestinal permeability and disrupted blood-brain barrier due to a decrease in the levels of tight junction proteins like Zonula occludens-1 (ZO-1) and E-cadherin (Wu et al., 2015). SOD1G93A mouse also shows reduced levels of butyrate-producing microbes like *Butyrivibrio fibrosolvens* and *peptostreptococcus* and enhanced pro-inflammatory cytokine IL-17 in the serum (Fang et al., 2016). Gut microbes are known to produce polyamines. Polyamines are naturally occurring low molecular weight polycations involved in a wide array of biological functions like cell proliferation, differentiation, gene regulation, and stress resistance and are known to be present in the vertebrate central nervous system (Seiler, 1985; Igarashi and Kashiwagi, 2010). The neuroprotective role of polyamines has been shown in the forebrain of gerbil and *Xenopus laevis* tadpoles epileptic model (Gilad and Gilad, 1991; Bell et al., 2011). In humans, polyamines are found in the form of putrescine, spermidine and spermine (Minois et al., 2011). Ornithine decarboxylase (ODC), an enzyme, catalyzes the decarboxylation of ornithine to create putrescine in the cytoplasm of cells. S-adenosyl-methionine decarboxylase and a transferase enzyme catalyze the transfer of the aminopropyl group to the main amine group of putrescine or spermidine, respectively, to create spermine and spermidine (Murray-Stewart et al., 2016). The enzyme ODC (putrescine biosynthesis III) can produce putrescine directly from L-ornithine or indirectly from L-arginine by the enzyme arginine decarboxylase (ADC; putrescine biosynthesis I and putrescine biosynthesis II) (Tabor et al., 1958; Tofalo et al., 2019). Spermidine is created by adding a propylamine moiety to putrescine, a process mediated by an enzyme known as spermidine synthase (Potter and Paton, 2014; Tofalo et al., 2019). The pathways for polyamine synthesis are shown in **Figure 6A**. The polyamines, including putrescine and spermidine, are affected in diseases like AD, PD, and MS (Morrison and Kish, 1995; Paik et al., 2010; Büttner et al., 2014; Bolayir et al., 2018; Makletsova et al., 2019). In AD, the levels of putrescine are increased by 70 percent. At the same time, spermidine concentration is decreased in the temporal cortex of AD patients (Morrison and Kish, 1995; Polis et al., 2021). In the case of PD, the concentrations of putrescine, N1-acetylspermidine, and putrescine spermidine-1 were found to be increased. In contrast, spermidine was significantly reduced in cerebrospinal fluid (CSF) of patients with PD and multiple system atrophy (MSA) (Paik et al., 2010). Since there are studies with evidence of microbes modulating the Gut-brain axis GBA by producing various neuroactive molecules in the human gut, the present study investigates the role of microbial dysbiosis in neurological disorders by analyzing the available microbial dysbiosis data from four neurodegenerative diseases. The potential of the change in microbial population regulating the production of neuroactive and neuro-immunomodulatory metabolites is analyzed. Insights from the current study will help understand the metabolic role of microbial dysbiosis in the pathogenesis and progression of neurodegenerative disorders.

## 2. Materials and methods

### 2.1. Extraction of microbial dysbiosis data in neurodegenerative diseases

**Figure 2:**
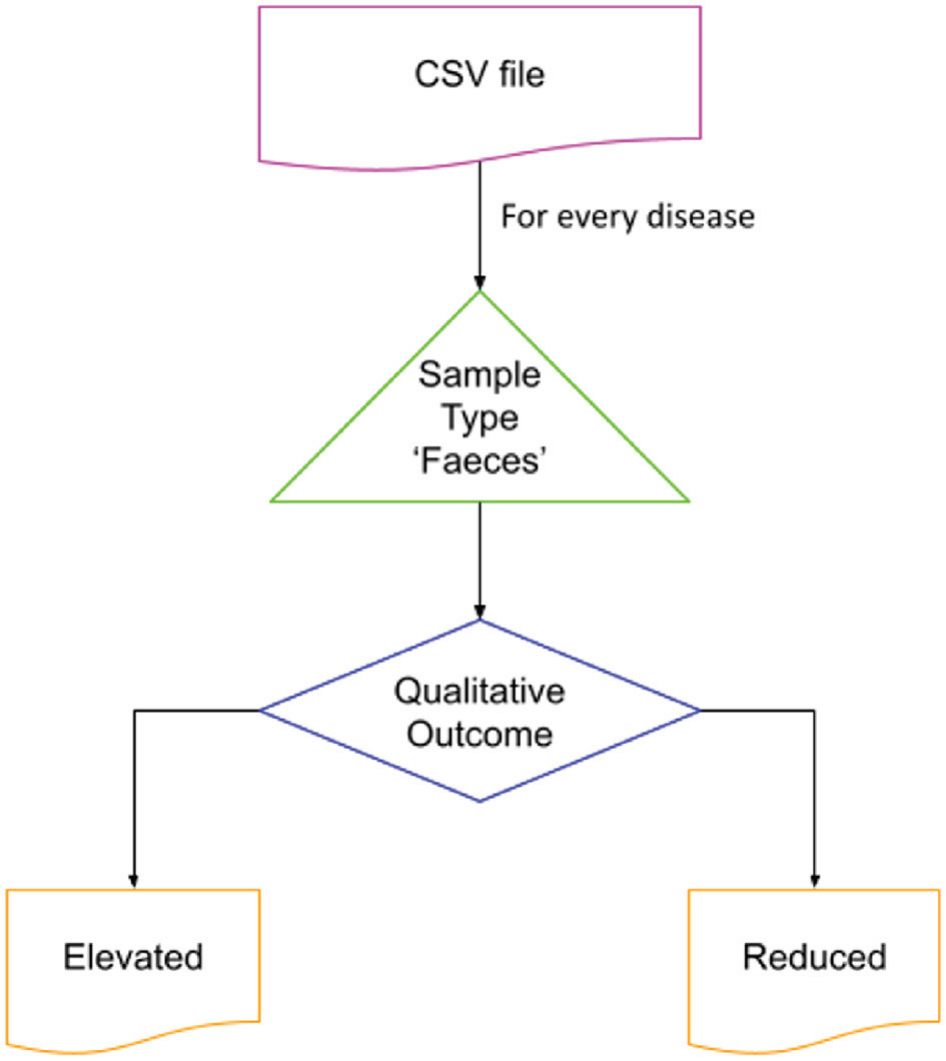
Extraction of microbial dysbiosis data from Disbiome database. The flow was iterated over the disease of interest for the sample type “Feces.” The list of elevated and reduced organisms reported was stored in separate files for further processing.

The Disbiome database was utilized to obtain disease-associated microbial data. This publicly accessible database provides information on changes in microbial composition in various diseased conditions (Janssens et al., 2018). The complete database records were exported through the Disbiome JSON web service, and a python script was used to extract data for the diseases of interest. The sample type “Feces” was selected to represent the gut microbial community. The script generated two output files identifying microbial populations that were elevated or reduced in connection with the condition of interest. Given the large number of diseases represented in the source data file, a python script was developed to preprocess and extract the data specific to the chosen diseases. The flow of code is shown in Figure 2.

### 2.2. Multiple microbial list analysis

Multiple lists of microbes in neurodegenerative diseases were compared using molbiotool (multiple list comparator) online, and common microbes were identified in elevated and reduced conditions (Molbiotools, 2023).

### 2.3. Taxonomic classification and phylogenetic tree construction

The list of microbes was taxonomically classified using the NCBI taxonomy tool, and the file was downloaded in phylip format (Federhen, 2012). The tree was viewed using NCBI phylogenetic tree viewer, and the output file was downloaded in Newick format (National Center for Biotechnology Information NCBI, 2023a,b). Phylogenetic tree construction was performed using an iTOL -interactive Tree of Life (Letunic and Bork, 2007).

### 2.4. Metabolic pathway analysis

The metabolic capacity of microbes was explored using MACADAM (MetAboliC Pathways Database for Microbial taxonomic groups) and its web browser MACADAMexplore (Le Boulch et al., 2019). MACADAM is a pathway genome database (PGDB) that links with the metabolic pathway database MetaCyc, which contains metabolic pathways and associated enzymes for a given taxon. We enquired about the associated metabolic pathways for the most significantly elevated and reduced microbes in specific neurodegenerative diseases. The Database file was accessed using the DB Browser for SQLite using the custom script provided by MACADAM. The Pathway Scores (PS) were calculated for each microbe by dividing the number of reactions annotated in the genome of that microbe by the total number of reactions constituting the particular pathway (**Figure 5B**). The equation for calculating the PS is as follows.


$$\begin{array}{l} \text{Pathway score (PS)}=\frac{\text{Number of reactions that have annotation in the genome for a pathway}}{\text{Total number of reactions constituting the pathway}} \end{array}$$

The range of PS, which represents the completeness of a metabolic pathway in an organism's genome, is from 0 to 1. A PS score of 1 means that all enzymes needed for the pathway are present in the organism's genome, while a PS score of 0 means that none of the required enzymes are in the organism's genome. The heatmap was created using a PS for the specific metabolic pathways in the group of microbes with elevated and reduced populations. The heatmap was made using MetaboAnalyst 5.0 with Euclidean distance measure and ward clustering with standardized auto-scale features (Pang et al., 2021).

## 3. Results

### 3.1. Microbial dysbiosis signatures differ in ALS compared to AD, PD, and MS

**Figure 3:**
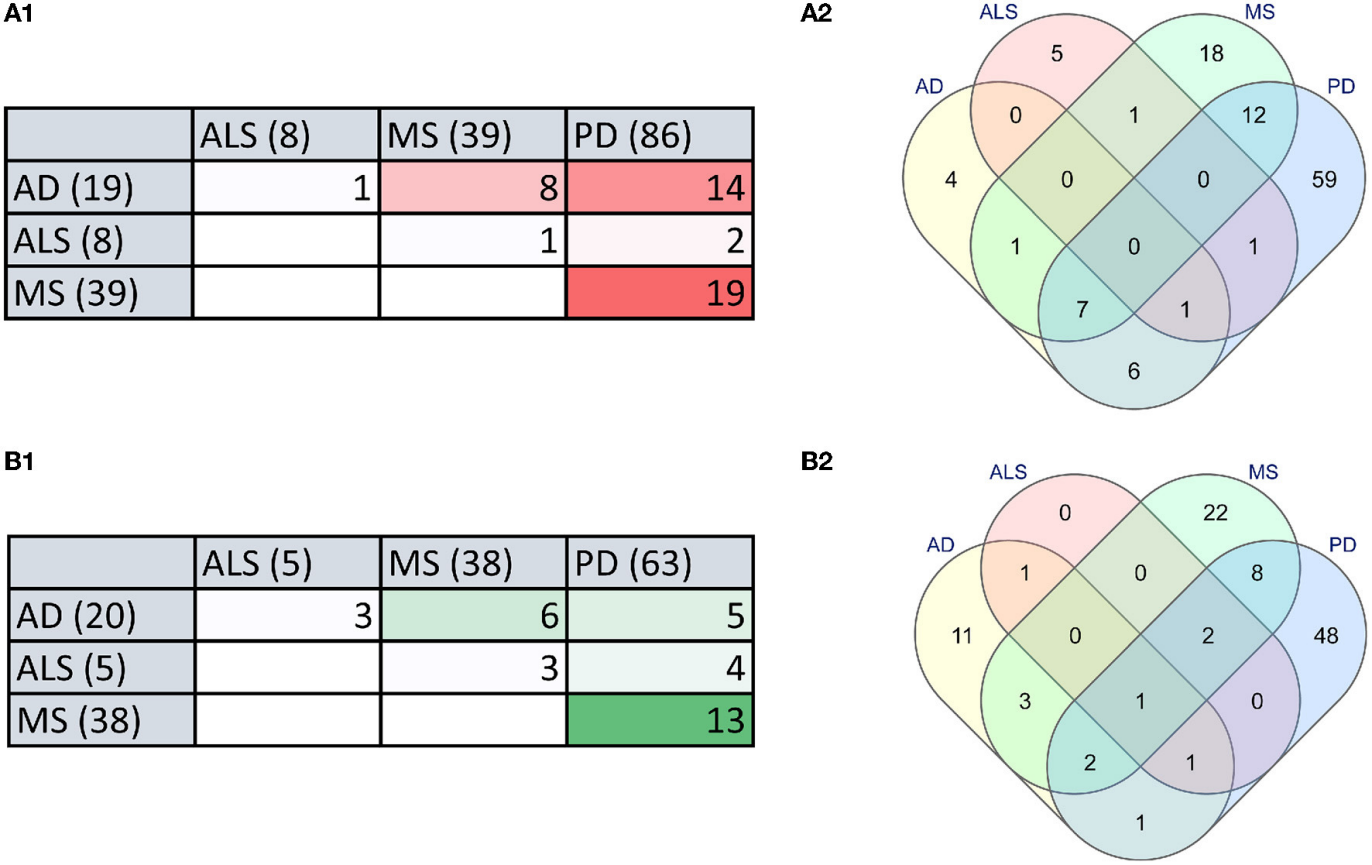
The microbial dysbiosis comparison in neurodegenerative diseases shows similarities in elevated and reduced groups of bacteria. **(A)** Comparison of elevated microbial population in AD, PD, MS, and ALS in the form of a Table **(A1)** and Venn diagram **(A2)**. **(B)** Comparison of reduced microbial population in AD, PD, MS and ALS in the form of a Table **(B1)** and Venn diagram **(B2)** (numbers in brackets indicate the total number of dysbiotic microbial genera reported in that disease).

To identify the relationship between microbial dysbiosis with neurodegenerative diseases, we compared the microbes with changes in population in four neurodegenerative diseases, AD, PD, MS, and ALS. Interestingly, microbial dysbiosis showed the highest similarity between the two diseases, MS and PD (Figures 3A,  B). In these two diseases, 19 and 13 identical genera of microbes were identified with an elevated and reduced population, respectively. Moreover, 14 identical genera of microbes in AD and PD had elevated populations. On the other hand, MS and AD had eight and six common microbe genera with an elevated and reduced population, respectively. Additionally, AD exhibited six and five microbe genera as common with reduced population along with MS and PD, respectively. In contrast, ALS appeared to be less associated with microbial dysbiosis compared to MS and AD, with only one microbe genus, *Methanobrevibacter* and *Ruminococcaceae* being commonly present under elevated conditions, respectively. Similarly, ALS had only three microbe genera in common in reduced population conditions with MS and AD. Also, two and four microbes were found to be similar between ALS and PD in elevated and reduced conditions, respectively (Figure 3). The most common microbes found in elevated and reduced conditions in these four diseases are shown in (Table 1).

**Table 1:** Commonly affected bacterial groups in Multiple sclerosis, Alzheimer's disease, Amyotrophic lateral sclerosis, and Parkinson's disease.

**Sr. no**.	**Organism**	**NCBI taxonomy id**	**Condition**	**Disease**	**MedDRA ID**	**Detection method**
1	*Alistipes*	239759	Elevated	Alzheimer's disease	10012271	16S rRNA sequencing
			Elevated	Multiple sclerosis	10028245	16S rRNA sequencing
			Elevated	Parkinson's disease	10061536	Shotgun sequencing
2	*Bacteroides*	29523	Elevated	Alzheimer's disease	10012271	16S rRNA sequencing
			Elevated	Multiple sclerosis	10028245	Metagenomic sequencing
			Elevated	Parkinson's disease	10061536	RNA gene amplicon sequencing
3	*Bifidobacterium*	41200	Elevated	Alzheimer's disease	10012271	16S rRNA sequencing
			Elevated	Multiple sclerosis	10028245	16S rRNA sequencing
			Elevated	Parkinson's disease	10061536	16S rRNA sequencing
4	*Blautia*	572511	Elevated	Alzheimer's disease	10012271	16S rRNA sequencing
			Elevated	Multiple sclerosis	10028245	16S rRNA sequencing
			Elevated	Parkinson's disease	10061536	RNA gene amplicon sequencing
5	*Lactobacillus*	1591	Elevated	Alzheimer's Disease	10012271	16S rRNA sequencing
			Elevated	Multiple Sclerosis	10028245	16S rRNA sequencing
			Elevated	Parkinson's Disease	10061536	qPCR
6	*Ruminococcaceae*	219	Elevated	Alzheimer's Disease	10012271	16S rRNA sequencing
			Elevated	Amyotrophic lateral sclerosis	10002026	16S rRNA sequencing
			Elevated	Parkinson's disease	10061536	16S rRNA sequencing
7	*Shigella*	625	Elevated	Alzheimer's disease	10012271	qPCR
			Elevated	Multiple sclerosis	10028245	16S rRNA sequencing
			Elevated	Parkinson's disease	10061536	16S rRNA sequencing
8	*Clostridium*	1506	Reduced	Alzheimer's disease	10012271	16S rRNA sequencing
			Reduced	Amyotrophic lateral sclerosis	10002026	16S rRNA sequencing
			Reduced	Multiple sclerosis	10028245	qPCR
			Reduced	Parkinson's disease	10061536	qPCR
9	*Blautia*	572511	Reduced	Alzheimer's disease	10012271	16S rRNA sequencing
			Reduced	Multiple sclerosis	10028245	16S rRNA sequencing
			Reduced	Parkinson's disease	10061536	RNA gene amplicon sequencing
10	*Dorea*	189330	Reduced	Alzheimer's disease	10012271	16S rRNA sequencing
			Reduced	Multiple sclerosis	10028245	16S rRNA sequencing
			Reduced	Parkinson's disease	10061536	RNA gene amplicon sequencing
			Reduced	Amyotrophic lateral sclerosis	10002026	16S rRNA sequencing
11	*Faecalibacterium*	216851	Reduced	Alzheimer's disease	10012271	16S rRNA sequencing
			Reduced	Multiple sclerosis	10028245	PhyloChip Array
			Reduced	Parkinson's disease	10061536	16S rRNA sequencing
12	*Lachnospiraceae*	186803	Reduced	Alzheimer's disease	10012271	16S rRNA sequencing
			Reduced	Multiple sclerosis	10028245	16S rRNA sequencing
			Reduced	Parkinson's disease	10061536	16S rRNA sequencing
13	*Ruminococcus*	41978	Reduced	Alzheimer's disease	10012271	16S rRNA sequencing
			Reduced	Multiple sclerosis	10028245	16S rRNA sequencing
			Reduced	Parkinson's disease	10061536	MiSeq sequencing

### 3.2. Dysbiosis in neurodegenerative diseases linked to decreased abundance of *Bacteroidetes* and *Firmicutes*

**Figure 4:**
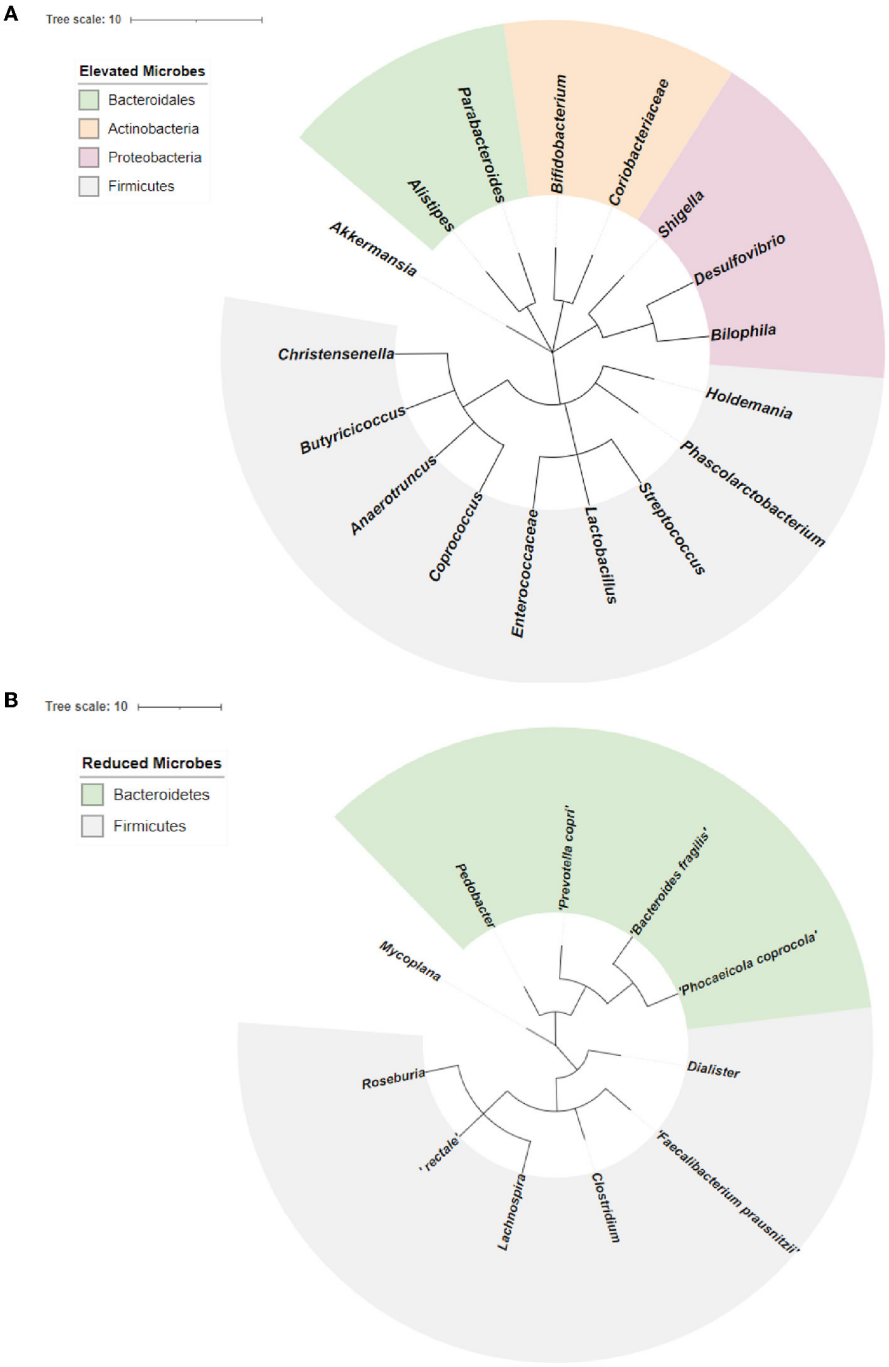
Phylogenetic analysis of overlapping microbial dysbiosis in diseases including AD, PD, MS, and ALS. **(A)** Phylogenetic tree of common microbes with the elevated population in AD, PD, MS, and ALS. **(B)** Phylogenetic tree of common microbes with the reduced population in AD, PD, MS, and ALS.

We performed a phylogenetic analysis of overlapping microbial dysbiosis between four major neurodegenerative diseases; AD, PD, MS, and ALS. All common microbes with elevated populations in these four diseases belonged to the phyla *Bacteroidetes, Actinobacteria, Proteobacteria*, and *Firmicutes*. In contrast, microbes with a reduced population majorly belonged to the phyla *Bacteroidetes* and *Firmicutes*. The most prominent microbial shift was reported from the phyla *Firmicutes* with an increase in the population of bacteria, including *Holdemania, Phascolarctobacterium, Streptococcus, Lactobacillus, Enterococcacease, Coprococcus, Anaerotruncus, Butyricicoccus*, and *Christensenelia*. These neurodegenerative diseases also showed a decrease in the population of *Pedobacter, Prevotella copri, Bacteroides fragilis*, and *Phocaeicola coprocola*, belonging to phyla *Bacteroidetes* (Figure 4).

### 3.3. Elevated microbes lacked capability to produce propionate and butyrate

**Figure 5:**
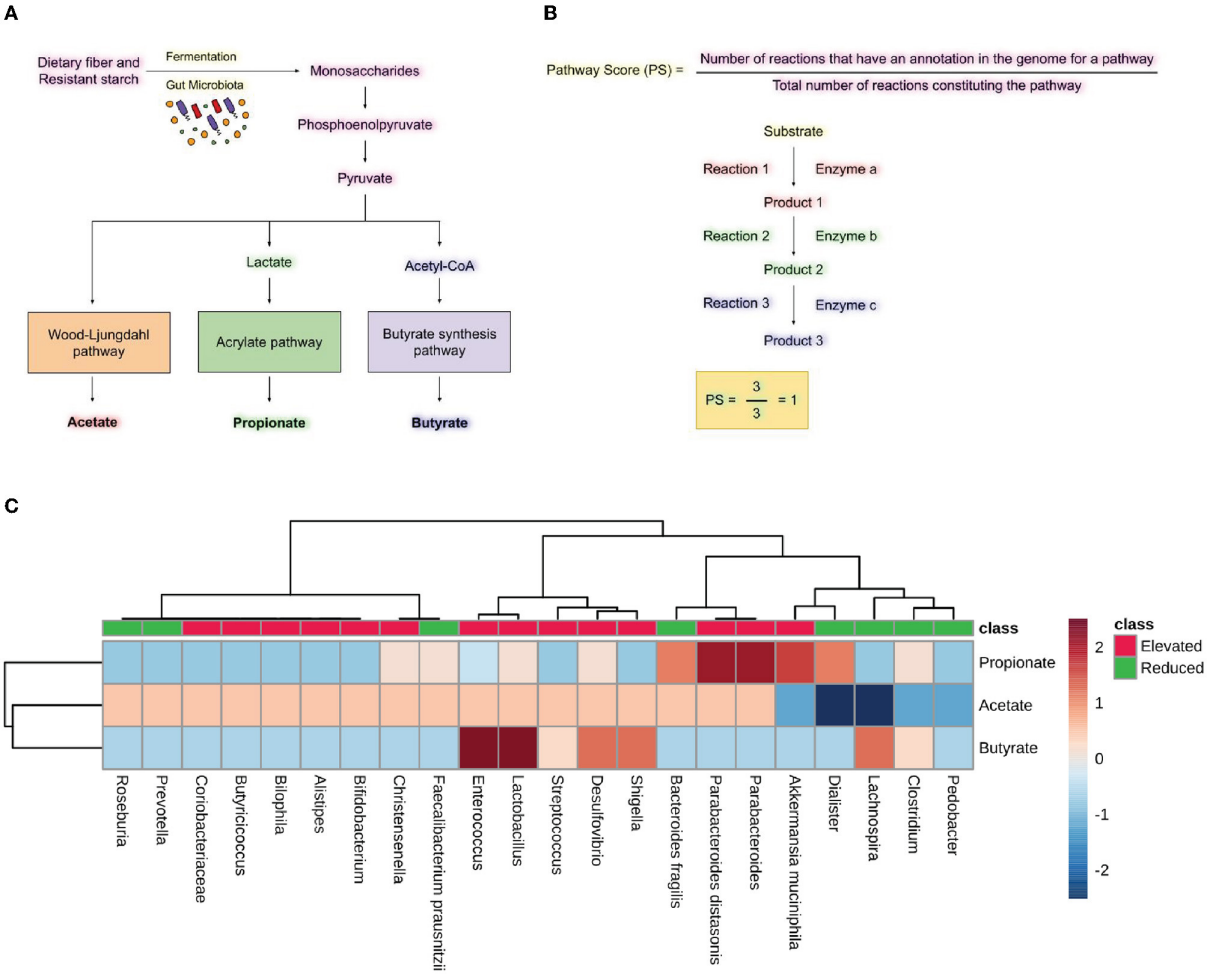
Short-chain fatty acid synthesis pathway analysis. **(A)** Pathway for producing SCFA by gut microbiota by utilizing dietary fiber and resistant starch. **(B)** Formulas for calculating pathway scores (PS) of a metabolic pathway present in the microbes. **(C)** Heatmap showing pathway scores for producing SCFA like acetate, propionate, and butyrate in microbes found in elevated and reduced conditions in AD, PD, MS, and ALS.

We analyzed the metabolic pathways responsible for producing the SCFAs synthesized by the bacteria found to have either increased or decreased population in neurodegenerative diseases, including AD, PD, MS, and ALS. PS was calculated as shown in Figure 5B. Analysis of the scores revealed the abundance of microbes expressing the pathways for synthesizing Propionate and butyrate in the gut of diseased individuals. On the other hand, the microbes with reduced populations showed lower PS for propionate and butyrate production. *Parabacteroides distasonis* and *Akkermansia muciniphila* have the highest PS for producing propionate through the fermentation of pyruvate. Also, *Enterococcus* and *Lactobacillus* had the highest PS for butyrate formation through the fermentation of pyruvate. *Desulfovibrio* and *Shigella* showed a high score for the formation of butyrate, which is found in an elevated amount in neurodegenerative diseases. *Lachnospira* was the only bacterial species which had a reduced population in the diseased state but had a high PS for butyrate formation (Figure 5C). Additionally, the formation of acetate, propionate and butyrate from L-lysine and succinate were also analyzed. However, no significant difference was seen in the elevated and reduced microbial populations (Supplementary Figure 1).

### 3.4. Elevated microbes lacked pathways for spermidine synthesis

**Figure 6:**
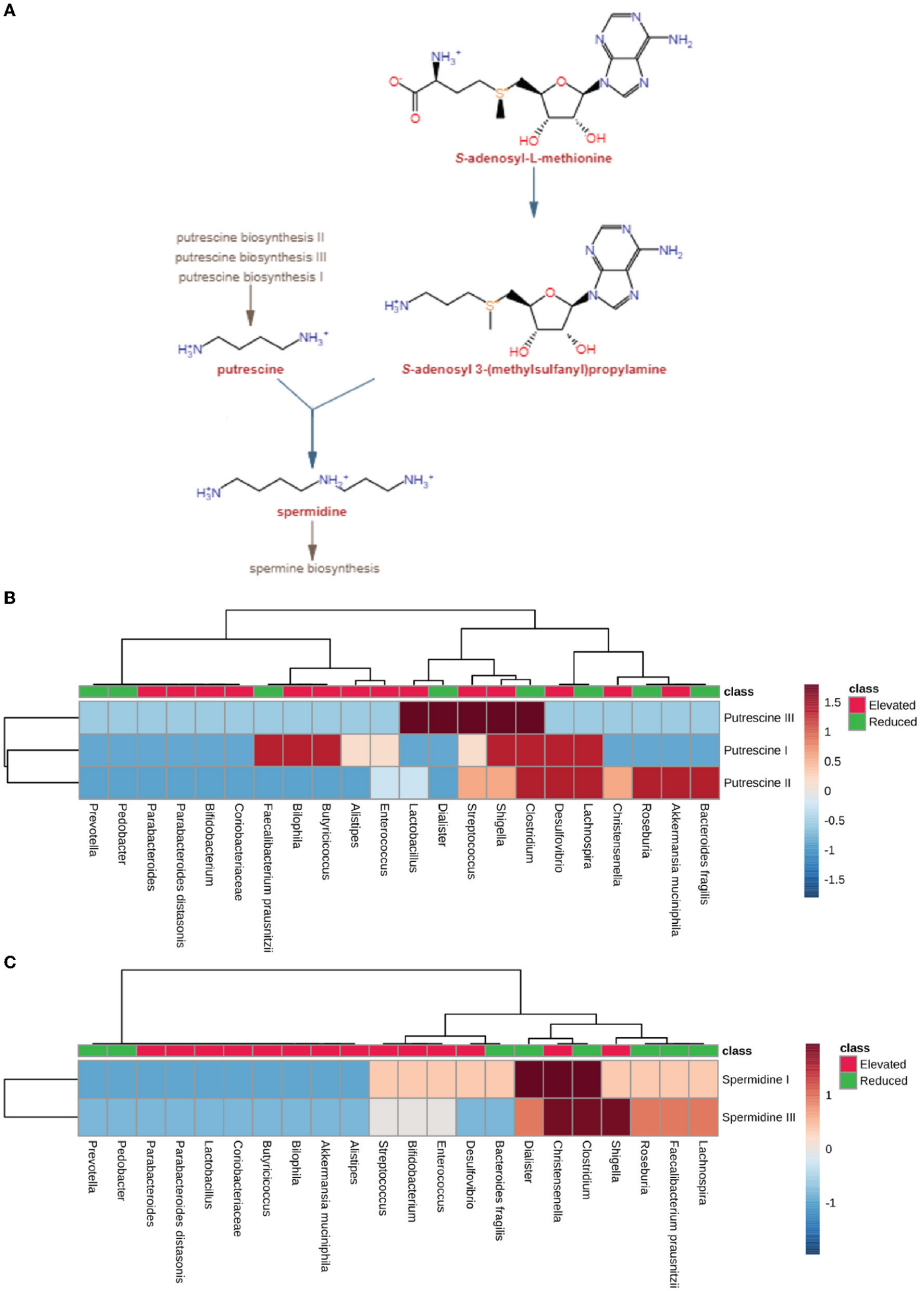
Polyamine synthesis pathway analysis. **(A)** Pathway for producing polyamine, including spermidine and putrescine, by using S-adenosyl-3(methylsulfonyl) as a precursor molecule by gut microbiota. **(B)** Heatmap showing pathway scores of putrescine synthesis in microbes found in elevated and reduced conditions in AD, PD, MS, and ALS. **(C)** Heatmap showing pathway scores of spermidine synthesis in microbes found in elevated and reduced conditions in AD, PD, MS, and ALS.

In order to understand the role of microbial dysbiosis with respect to the levels of polyamines in AD, PD, MS, and ALS, we checked the biosynthesis PS of microbes with an elevated or reduced population in these four diseases. The microbes with elevated populations showed higher PS for producing putrescine (Figure 6B). *Shigella, Lactobacillus*, and *Streptococcus* are such microbes with elevated populations and the highest PS for the putrescine III biosynthetic pathway. The microbes with elevated populations, like *Bilophila, Butryicicoccus, Alistipes, Enterococcus, Lactobacillus, Streptococcus, Shigella*, and *Desulfovibrio*, have the metabolic capability to produce putrescine majorly by the putrescine III biosynthesis pathway. *Streptococcus, Shigella, Desulfovibrio, Christensenelia*, and *Akkermansia muciniphila* have the putrescine III synthesis pathway and belong to the elevated microbes group. In contrast, *Clostridium, Lachnospira, Roseburia*, and *Bacteroides* were the exceptions from the reduced category of microbes. Overall, we observed low PS for spermidine biosynthesis in microbes with elevated populations in neurodegenerative disorders. Only *Christensenalia* has the highest PS for spermidine I and spermidine III synthesis pathway (Figure 6C). On the other hand, *Dialister* and *Clostridium* belonged to the reduced group and had a high PS for the spermidine I biosynthesis pathway.

### 3.5. Elevated microbes exhibited an absence of tryptophan and histamine synthesis pathways

**Figure 7:**
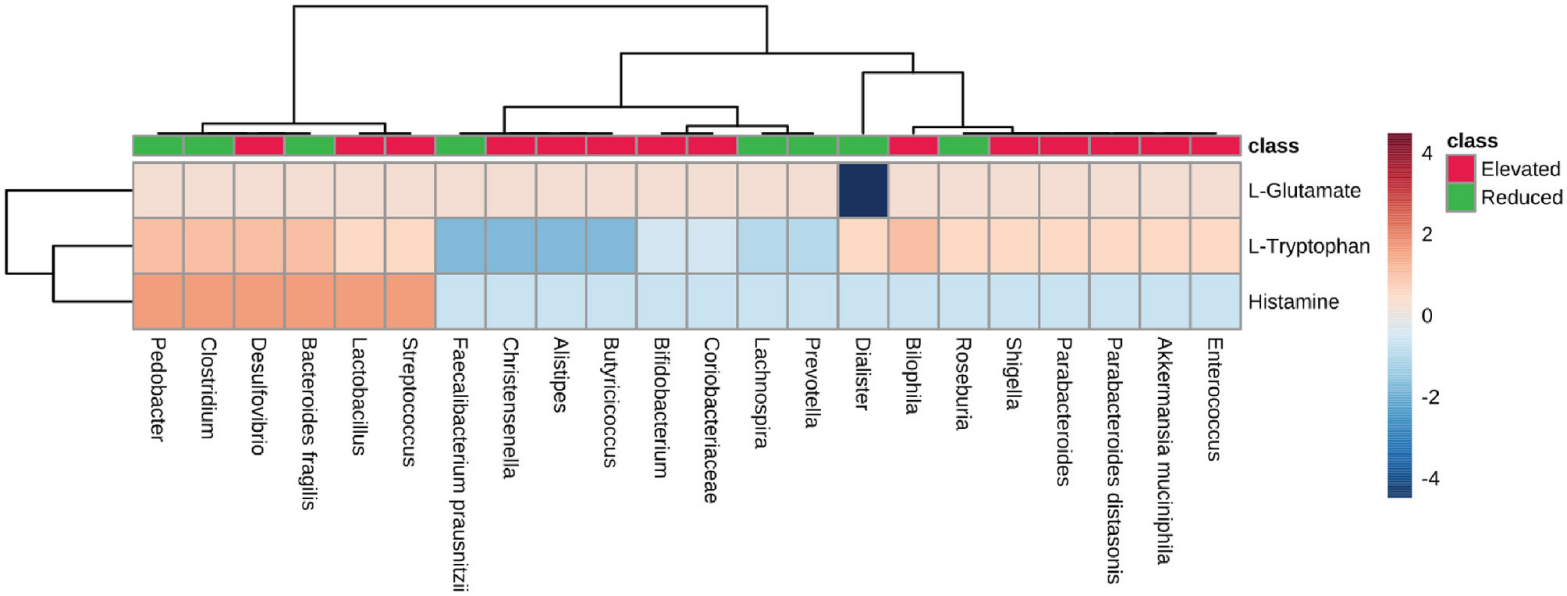
Neurotransmitter precursor synthesis pathway analysis. Heatmap showing pathway scores for producing neurotransmitter precursors like L-glutamate, L-Tryptophan, and histamine in the gut by microbes found in elevated and reduced conditions in AD, PD, MS, and ALS.

We analyzed the neuroactive compounds synthesis pathways with respect to microbial dysbiosis in AD, PD, MS, and ALS. First, we checked the pathway for glutamate synthesis, an important excitatory neurotransmitter required for neural communication and is present at the synapse of nerve cells. Glutamate is also a metabolic precursor for the synthesis of GABA (Reubi et al., 1978; Petroff, 2002; Waagepetersen et al., 2007). We found that the pathway for L-glutamate synthesis is present among most microbes (PS-0.33). The *Dialister*, which represents the group of microbes with a reduced population, exhibited no metabolic capacity to produce L-glutamate (Figure 7). Consequently, we looked at the tryptophan metabolic pathway, a precursor molecule for serotonin synthesis in the human gut (Kaur et al., 2019; Averina et al., 2020). The microbes with an elevated population showed mixed results concerning the PS for L-tryptophan synthesis. *Desulfovibrio* and *Bilophila* had high pathway scores among the group of microbes with an increase in population. On the other hand, *Pedobacter, Clostridium*, and *Bacteroides fragilis* belonged to the group with a reduced population and had a high PS for tryptophan synthesis. Apart from these microbes, *Fecalibacterium prausnitzii, Christensenella, Alistipes*, and *Butyricoccus* from the elevated microbes have shown no enzymatic capacity for synthesizing Tryptophan in diseased individuals (Figure 7). Histamine plays a vital role as a neurotransmitter in the CNS. Therefore, we analyzed the pathways for histamine synthesis with respect to microbial dysbiosis in these neurodegenerative diseases. Most of the microbes with elevated populations lacked the pathway responsible for histamine synthesis. However, *Desulfovibrio, Lactobacillus*, and *Streptococcus* exhibited the pathway for histamine synthesis and had an elevated population in the disease's conditions. The group with the reduced population, *Pedobacter, Clostridium*, and *Bacteroides fragilis*, were seen to have high pathway scores for histamine synthesis (Figure 7).

## 4. Discussion

**Figure 8:**
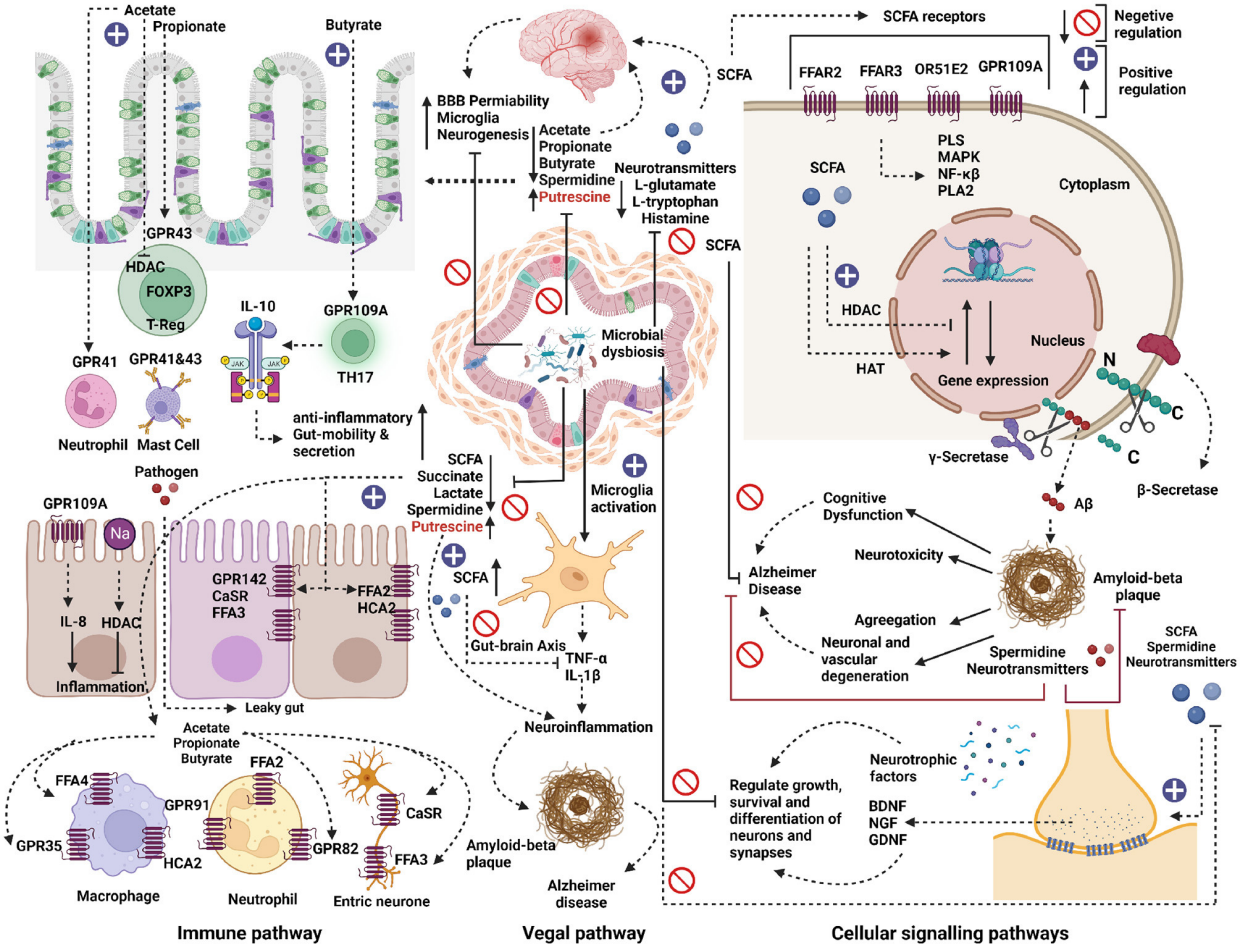
Metabolic role of microbial dysbiosis in neurodegenerative diseases. The elevated microbe in neurodegenerative diseases lacks a pathway for synthesizing the neurotransmitter precursors and neuromodulator metabolites. The enzymatic genes for synthesizing short-chain fatty acids (SCFA) like acetate and butyrate were less expressed in the genome of microbes found in elevated conditions in AD, PD, and ALS. SCFAs directly or indirectly influence gut-brain communication and brain function *via* immune, vagal, and cellular signaling pathways. SCFA interact with intestinal epithelial cells and immune cells, such as macrophages, T-cells, and neutrophils, by triggering free fatty acid receptors (FFARs) or hindering histone deacetylases. SCFAs help to maintain anti-inflammation, gut mobility, and secretion. Microbial dysbiosis directly or indirectly down-regulates the synthesis of SCFAs by inhibiting the SCFAs biosynthesis pathways and thus can contribute to its effect on the development of several neurological disorders. SCFAs can directly activate vagal afferents *via* FFARs. Also, due to microbial dysbiosis, microglia cells might get activated and release pro-inflammatory cytokines such as TNF-α and IL-1β, which can cause neuro-inflammation by forming amyloid-beta plaque and can trigger Alzheimer's disease. On the other hand, the elevated microbes lack pathways for synthesizing neurotransmitter precursor molecules like L-glutamate, L-tryptophan, and Histamine. The lack of these precursors can negatively affect the neuron's growth, survival, and differentiation of synapses. The neuroprotective compounds like spermidine were less expressed in the elevated microbes, which might result in neurotoxicity, neuronal, and vascular degeneration and cognitive dysfunction in neurodegenerative diseases.

The present study provides a comprehensive catalog of microbial dysbiosis and its potential metabolic involvement in neurodegenerative diseases, including AD, PD, MS, and ALS. The analysis showed a high similarity in the group of microbes found with elevated and reduced populations among these four neurogenerative diseases. Our comparative analysis indicated that ALS is quite different from AD, PD, and MS in terms of the presence and absence of certain microbes in these diseases. Phylogenetic analysis showed that the microbes commonly found with an elevated population in the four neurogenerative diseases belong to *Firmicutes, Bacteriodales, Actinobacteria*, and *Proteobacteria*. On the contrary, the microbes found with a reduced population were mainly represented by the phyla *Firmicutes* and *Bacteroidetes*. We were interested in uncovering the dysbiotic microbes' metabolic capabilities in these neurodegenerative diseases. Among many neuroactive metabolites, SCFA is predicted to affect brain function through multiple pathways like (1) modulation of neuroinflammation, (2) Blood-Brain Barrier (BBB) maintenance, (3) Brain metabolism regulation, (4) Epigenetic regulation, and (5) Interference in protein aggregate formation. The microbes which were most commonly found with an increased population in these neurodegenerative diseases lack the pathways for producing butyrate and propionate. The positive effects and association of reduced concentration of butyrate and propionate have been shown in animal studies in AD, PD, ALS, and MS. For instance, the Apolipoprotein E (APOE) transgenic mice (overexpressing human APOE3 or APOE4 gene), predisposed to developing AD and dementia, are shown to have decreased population of butyrate-producing gut microbiota (Tran et al., 2019). Interestingly, the administration of butyrate in the ALS mice model (G93A) alleviated the disease symptoms. Moreover, it increased the lifespan of the mutant mice (Zhang et al., 2017). Another study shows that MS patients exhibited reduced serum and fecal concentrations of propionic acid as compared to the control group. Additionally, administration of propionic acid in MS patients showed increased functionality of competent regulatory T cells and reduced brain atrophy (Duscha et al., 2020). SCFAs are known to interact with intestinal epithelial cells and immune cells by triggering free fatty acid receptors (FFARs) or hindering histone deacetylases, thereby helping to maintain anti-inflammation, gut mobility, and secretion (Chen et al., 2003). Altogether, this indicates that the increase in the population of microbes that lack the capability to produce beneficial SCFAs, such as butyrate and Propionate, might contribute to altered brain pathology and neurodegenerative disease progression. The current study's most exciting outcome is the metabolic capability of the dysbiotic microbial population to affect polyamine synthesis in these neurodegenerative diseases. We observed an increased population of microbes that lacks the pathway for producing spermidine. However, some of the microbes with an elevated population, like *Lactobacillus, Streptococcus*, and *Shigella*, had high pathway scores for producing putrescine through the putrescine 1 biosynthesis pathway. These results correlate well with previous studies where the concentrations of putrescine and spermidine have been shown to be affected in diseases like AD, PD, and MS (Morrison and Kish, 1995; Paik et al., 2010; Bolayir et al., 2018; Makletsova et al., 2019). In the case of Alzheimer's, the level of putrescine is increased to 70%. At the same time, spermidine concentration is decreased in the temporal cortex of AD patients because of Polyamine Stress Response (PSR) (Morris et al., 2017; Polis et al., 2021). In Parkinson's disease, the concentrations of putrescine, N1-acetylspermidine, and putrescine spermidine-1 are increased. In contrast, spermidine is significantly reduced in cerebrospinal fluid CSF of Parkinson's disease (PD) and multiple system atrophy (MSA) patients (Paik et al., 2010). Overall, this suggests that microbial dysbiosis in neurodegenerative disorders can be linked with a reduced population of microbes with an inability to produce neuroprotective compounds like spermidine in the human gut. In addition, our results provide a glimpse of altered neurotransmitter precursor synthesis in the gut of diseased individuals. For example, the L-glutamate biosynthesis pathway, an essential excitatory neurotransmitter molecule and a precursor molecule for GABA synthesis are present in microbes, both with an elevated and reduced population, in the diseased individuals. These findings correlate with studies which suggest that extracellular glutamate leads to excitotoxicity *in vitro* and *in vivo* in astrocytes and neurons through the overactivation of ionotropic glutamate receptors (Meldrum, 1994; Lewerenz and Maher, 2015); The role of glutamate in epilepsy and other CNS disorders (Wikinski and Acosta, 1995). Chronic excitotoxicity is hypothesized to play a role in numerous neurodegenerative disorders, including AD, ALS, and Huntington's disease (Coyle and Schwarcz, 1976; De Belleroche et al., 1984; Basun et al., 1990; Beal et al., 1992; Carriedo et al., 1996; Araki et al., 2001; Talantova et al., 2013). Notably, many microbes with elevated populations cannot produce L-tryptophan, a necessary metabolic precursor for serotonin synthesis. The metabolism of tryptophan also leads to the production of six neuroactive compounds altogether called TRYP-6, reported affecting the Gut-Brain Axis (GBA) (O'Mahony et al., 2015; Kaur et al., 2019). These six metabolites include indole-3-acetic acid (IAA), quinolinate, kynurenine, indole, tryptamine and indole propionic acid (IPA) (Agus et al., 2018; Roager and Licht, 2018). The findings correspond with the belief that 5-HT levels are associated with serotonin in the pathogenesis of PD, ALS, and MS (Davidson et al., 1977; Dorszewska et al., 2013). Moreover, histamine and other important neurotransmitters were synthesized in reduced amounts by the microbes with an elevated population in the diseased state. Most of the microbes, except *Desulfovibrio, Lactobacillus*, and *Streptococcus*, showed no annotation in their genome to synthesize histamine. Initially, histamine was recognized as a neurotransmitter, although recent evidence supports its role as a peripheral inflammatory mediator and modulatory role in innate immune response (Rocha et al., 2014). Histamine has a dual role in modulating neuronal survival in neurodegenerative diseases, including AD, PD, and MS (Shan et al., 2012; Rocha et al., 2014). The lack of these precursors can negatively affect the neuron's growth, survival, and differentiation of synapses. Neuronal degeneration in the diseased state may lead to the degranulation of mast cells resulting in histamine release in the Cerebrospinal fluid (CSF) and increased BBB permeability. This, in turn, modulates the release of nitric oxide and other inflammatory mediators by the microglia cells. Subsequently, upon activation, the microglia secretome leads to dopaminergic neuronal death (Rocha et al., 2014). Microglia cells activation and release of pro-inflammatory cytokines such as TNF-α and IL-1β, which can cause neuro-inflammation by forming amyloid-beta plaque and might trigger pathogenesis in Alzheimer's disease. Overall, this study suggests the potential role of microbial dysbiosis in the altered concentration of histamine in diseased individuals. The current analysis represents a holistic view of the microbial dysbiotic community affecting brain function by synthesizing various neuroactive and neuro-immunomodulatory compounds. Further wet-lab experimentation is required to establish the specific role of microbes at the individual and population levels in these neurodegenerative pathology and disease progression (Figure 8).

## 5. Conclusion

In this study, we provide a comprehensive understanding of microbial dysbiosis and its metabolic potential in neurodegenerative diseases. By performing a comparative *in silico* analysis of gut microbial dysbiosis data from patients with AD, PD, MS, and ALS, we found a striking similarity in microbial dysbiosis signatures between AD, PD, and MS, while ALS had a different microbial profile. The elevated populations of microbes in neurodegenerative diseases lacked butyrate and propionate synthesis pathways, and we propose microbial dysbiosis as a possible link to altered concentrations of SCFA in diseased individuals. Our results also indicate imbalances in neurotransmitter precursor synthesis, with decreased representation of tryptophan and histamine and lower representation of the neuroprotective compound spermidine in the genome of microbes with an elevated population. These insights can aid future experiments to enhance our understanding of microbial dysbiosis and its role in neurodegenerative diseases, leading to microbiome-based therapeutic and diagnostic approaches.

## Data availability statement

The datasets presented in this study can be found in online repositories. The names of the repository/repositories and accession number(s) can be found in the article/Supplementary material.

## Ethics statement

Ethical review and approval was not required for the study on human participants in accordance with the local legislation and institutional requirements. Written informed consent for participation was not required for this study in accordance with the national legislation and the institutional requirements.

## Author contributions

VC: study conception and design, experimental operation, data analysis, and writing-original drafts. NC: study conception, data extraction, and analysis. SD: experimental operation and writing-reviewing and editing. DP: writing-reviewing and editing. UN: supervision and guidance. All authors contributed to the article and approved the submitted version.
